# Construction of FeCo_2_O_4_@N-Doped Carbon Dots Nanoflowers as Binder Free Electrode for Reduction and Oxidation of Water

**DOI:** 10.3390/ma13143119

**Published:** 2020-07-13

**Authors:** Aniruddha Kundu, Akhmad Irhas Robby, Arnab Shit, Hyeong Jun Jo, Sung Young Park

**Affiliations:** 1 Department of Chemical and Biological Engineering, Korea National University of Transportation, Chungju 380-702, Korea; write2aknow@gmail.com (A.K.); arnabshit@yahoo.in (A.S.); jhg4629@ut.ac.kr (H.J.J.); 2 Department of Green Bio Engineering, Korea National University of Transportation, Chungju 380-702, Korea; ahmadir93.ai@gmail.com; 3 Department of IT Convergence, Korea National University of Transportation, Chungju 380-702, Korea

**Keywords:** water splitting, carbon dots, iron-cobalt oxide, hydrogen evolution reaction, oxygen evolution reaction

## Abstract

Electrochemical water splitting is known as a potential approach for sustainable energy conversion; it produces H_2_ fuel by utilizing transition metal-based catalysts. We report a facile synthesis of FeCo_2_O_4_@carbon dots (CDs) nanoflowers supported on nickel foam through a hydrothermal technique in the absence of organic solvents and an inert environment. The synthesized material with a judicious choice of CDs shows superior performance in hydrogen and oxygen evolution reactions (HER and OER) compared to the FeCo_2_O_4_ electrode alone in alkaline media. For HER, the overpotential of 205 mV was able to produce current densities of up to 10 mA cm^−2^, whereas an overpotential of 393 mV was needed to obtain a current density of up to 50 mA cm^−2^ for OER. The synergistic effect between CDs and FeCo_2_O_4_ accounts for the excellent electrocatalytic activity, since CDs offer exposed active sites and subsequently promote the electrochemical reaction by enhancing the electron transfer processes. Hence, this procedure offers an effective approach for constructing metal oxide-integrated CDs as a catalytic support system to improve the performance of electrochemical water splitting.

## 1. Introduction

Electrochemical water splitting has attracted extensive interest as a desirable method to produce renewable energy in the form of hydrogen fuels (H_2_) [1,2,3,4]. Water splitting is mainly accompanied by a hydrogen evolution reaction (HER) which is thermodynamically feasible [5,6] and an oxygen evolution reaction (OER) which interferes in the water splitting process due to sluggish four proton-coupled electron transfer [7]. Hence, in order to overcome the high activation barrier of OER, a large overpotential (*η*) is needed in order to practically produce H_2_ [8]. Currently, catalysts based on noble metals such as IrO_2_, RuO_2_ and Pt are widely applied to support hydrogen evolution (cathode) and oxygen evolution (anode) reactions because they can provide high current densities [9,10,11,12,13]. However, these catalysts are expensive, rare and have an unstable catalytic performance. Therefore, developing low-cost, highly efficient and stable electrolyzers from earth-abundant metals is necessary in order to enhance hydrogen production. In recent years, scientists have devoted themselves to developing earth-abundant metals as alternative electrocatalysts, including oxides/hydroxides [14,15,16,17], chalcogenides [18,19,20,21], phosphates [22,23,24], phosphides [25,26,27], perovskite solids [28,29,30], carbides [31], borates [32], etc., for efficient OERs and HERs with suitable overpotentials.

FeCo-based metal nanocomposite has been widely investigated for energy storage functions, especially for batteries or supercapacitors [33,34,35,36]. Fe^3+^ addition into metal oxides or hydroxides is capable of reducing the overpotential for OER [37,38,39]. Several reports such as Yan et al. [40] have designed a FeCo_2_O_4_ coupled with graphene oxide spheres to improve the electrocatalytic activities of the oxygen evolution reaction. The combination of FeCo_2_O_4_ with nitrogen-enriched porous carbon was also reported to provide efficient water oxidation [41]. For improving electrocatalytic performance, the increase in the active site number and reactivity becomes a key factor in the catalytic process. Recently, a facile strategy to design an OER with more active sites was reported by using iron-cobalt oxide nanosheets [42].

To improve catalytic performance, carbon nanomaterials are usually combined with metal-based electrocatalysts [43,44,45]. For example, graphitic carbon nitrides (g-C_3_N_4_) have been presented in the field of photocatalytic water splitting for hydrogen production [46,47]. Carbon dots (CDs) with a diameter of <10 nm possess unique electron-transfer abilities and a large specific surface area, rendering them excellent catalysts as well as potential candidates for versatile applications [48,49,50,51,52,53,54,55,56,57]. The presence of several functional groups (-OH, -COOH, -NH_2_, etc.) on the surface of CDs provides a lot of sites for electroactive catalyst fabrication, and the existence of organic groups also affects the surface wettability. This phenomenon will improve the interface area between metal components, CDs and electrolytes, which will assist the kinetics of electrochemical reactions. Compared to other carbon materials, CDs are known to be more flexible, enabling them to form multicomponent nanostructures as well as render more exposed active sites when assembled with binary metal components. The interactions inside these nanostructures can promote intermolecular electron transfer, which is crucial in HERs. Hence, the integration of CDs will provide a large surface area to enhance the catalytic performance of binary metal oxide, and these advantages make CDs very promising in the electrocatalytic field.

Inspired by the above findings, we have successfully prepared CDs integrating FeCo_2_O_4_ nanostructures supported on three-dimensional (3D) Ni foam. We have studied the electrocatalytic activity of different electrocatalysts towards OER and HER under alkaline electrolytes and examined the electrochemical durability in order to determine the effects of CDs on the electrochemical phenomenon.

## 2. Materials and Methods

### 2.1. Materials

Iron nitrate nonahydrate (Fe(NO_3_)_3_·9H_2_O), cobalt nitrate hexahydrate (Co(NO_3_)_2_·6H_2_O), ammonium fluoride (NH_4_F), urea (CO(NH_2_)_2_), anhydrous ethanol (CH_3_CH_2_OH) and poly(vinyl pyrrolidone) (PVP, *M_w_* = 55,000) were purchased from Sigma Aldrich, Korea. Hydrochloric acid (HCl), potassium hydroxide (KOH) and sodium hydroxide (NaOH) were purchased from Samchun Chemicals, Korea. Ni foam with a 1.6 mm thickness was purchased from Alantum, Korea. Deionized (DI) water and double-distilled water (DDW) were used especially for the purposes of preparing solutions and washing.

### 2.2. Characterizations

The particle sizes of CDs were measured using dynamic light scattering (DLS) spectroscopy (Zetasizer Nano, Malvern, Germany). Infrared spectroscopy was performed using the Fourier-transform infrared (FT-IR) spectrometer (Thermoscientific, Nicolet 380, Seoul, Korea). The diffraction pattern of the sample was determined using X-ray diffraction (XRD; Bruker AXS D-8 ADVANCE, Cu *Kα* radiation). The morphology and microstructural features were observed using field-emission scanning electron microscopy fitted with energy dispersive spectroscopy (FESEM-EDS, JEOL JSM-6700F, Tokyo, Japan) and high-resolution transmission electron microscopy (HRTEM; JEOL JEM-2100F, Tokyo, Japan). The chemical compositions of the samples were assessed using X-ray photoelectron spectroscopy (XPS, Omicrometer ESCALAB, Taunusstein, Germany). The binding energy environment was evaluated with a monochromatic Al*-Kα* source at *hν* = 1486.6 eV, with an X-ray power of 25.6 W. The calibration of the binding energies was performed for specimen charging. The C 1s peak at 284.6 eV was taken as a reference and the values are accurate to ±0.1 eV. The surface area of the sample was calculated using the Micromeritics 3Flex instrument, following the Brunauer–Emmett–Teller (BET) process by using N_2_ adsorption–desorption.

### 2.3. Synthesis of N-Doped Carbon Dot (CD)

The CD was synthesized from the carbonization of PVP polymer through the hydrothermal process. PVP is a precursor for synthesizing CD, which also becomes the nitrogen source for doping. Briefly, PVP polymer (1 g) was dissolved in DDW (50 mL) and placed in a Teflon-lined autoclave for hydrothermal processing in an electric oven (180 °C, 8 h). The solution was further frozen and dried in a freeze dryer (ilShinBioBase, Model No. FD8508, Dongducheon, Korea) to obtain CD powder, with an obtained yield of 91.28%.

### 2.4. Synthesis of CDs-FeCo_2_O_4_ Nanohybrid Supported on Ni Foam

Ni foam (2 × 4 cm) was immersed in dilute HCl and cleaned ultrasonically with DI water and ethanol to remove the oxide layer on the surface, followed by further drying overnight in an oven at 60 °C. For synthesis, Fe(NO_3_)_3_·9H_2_O (1 mmol), Co(NO_3_)_2_·6H_2_O (2 mmol), and different amounts of CD powder (5, 25 and 40 mg) were mixed in DI water (60 mL), with the consecutive addition of NH_4_F (5 mmol) and CO(NH_2_)_2_ (10 mmol) under stirring. The solution was then transferred into a Teflon-lined autoclave, followed by inserting the cleaned Ni foam and placing the sealed autoclave in an electric oven for the hydrothermal processing (140 °C, 8 h). After completion of the reaction, Ni foam with grown Fe-Co precursor was taken out, washed vigorously with DDW and dried overnight at 60 °C. Subsequently, the sample was annealed at 400 °C for 2 h (heating rate: 2 °C min^−1^) to obtain the FeCo_2_O_4_@N-doped CD nanohybrid. For comparison, FeCo_2_O_4_ was also synthesized using the same conditions but without the addition of CDs. The prepared samples were denoted as FC for FeCo_2_O_4_ and FCCD5, FCCD25 and FCCD40 for FeCo_2_O_4_ doped with 5 mg, 25 mg and 40 mg of CDs, respectively.

### 2.5. Electrochemical Measurements

An electrochemical work station (CS350, CorrTest Instrument, Wuhan, China) equipped with a three-electrode cell system (reference electrode: Hg/HgO; counter electrode: Pt wire; working electrode: materials are grown on 1 cm^2^ Ni foam) was used for electrochemical measurements at room temperature using an alkaline electrolyte (KOH 1 M). The polarization curves of HER and OER were obtained using linear sweep voltammetry (LSV, scan rate: 5 mV s^−1^), whereas the stabilities were measured using a cyclic voltammetry test (CV, scan rate: 50 mV s^−1^). Chronopotentiometric studies were conducted to test the electrochemical durability of the samples with a constant current density (10 mA cm^−2^ for HER, 50 mA cm^−2^ for OER). To estimate the electrochemical double-layer capacitance (*C_dl_*), CV was performed under different scan rates, and the electrochemically active surface area (ECSA) was calculated based on *C_dl_* values. The Tafel slopes were calculated from the polarization curve according to the Tafel equation:

*η* = *a* + *b* log *j* (1)

where *η* is the overpotential, *a* is the intercept, *b* is the Tafel slope and *j* is the current density. Electrochemical impedance spectroscopy (EIS) was conducted at a frequency of 10^5^ to 0.1 Hz. An equivalent RC circuit was utilized to fit the impedance spectra, and the semicircle diameter of the Nyquist plot was used to determine the charge transfer resistance (*R_ct_*). The measured potentials were transformed into a reversible hydrogen electrode (RHE) via RHE calibration using the Nernst equation:

*E*_RHE_ = *E*_Hg/HgO_ + 0.098 V + 0.059 pH (2)

For the Mott–Schottky plot, the impedance results were collected in the potential range of −1.2 to 1.5 V.

## 3. Results and Discussion

**Figure 1:**
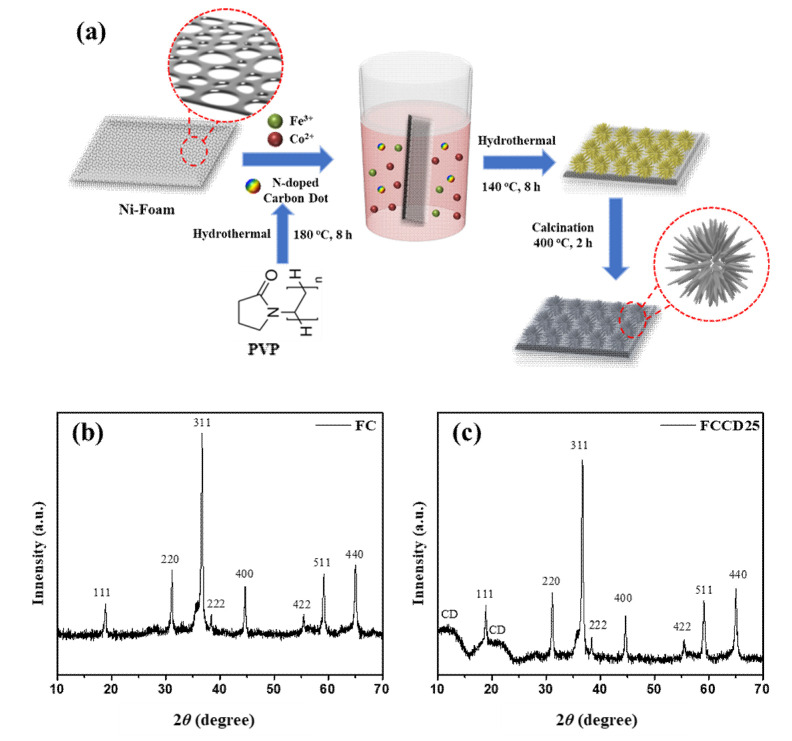
(**a**) Schematic description for the synthesis of FeCo_2_O_4_@CDs nanoflowers, XRD pattern for (**b**) FC and (**c**) FCCD25.

The synthesis process of nanostructured FeCo_2_O_4_@N-doped CDs (FCCD) is delineated in Figure 1a. An Fe-Co precursor was initially synthesized by a facile hydrothermal technique using the aqueous solution of respective metal salts, urea, NH_4_F and different amounts of CDs. Then, the Fe-Co precursor was thermally transformed into spinel FeCo_2_O_4_ via controlled calcination in air at 400 °C to obtain the product grown on Ni foam. During this calcination process, H_2_O and CO_2_ gases are released and a large number of pores are created within FeCo_2_O_4_. The major benefit of the hydrothermal method is the uniform growth of the microstructure, even in complex architectures containing various dopants and elements, without altering the homogeneity of the structure and morphology. The nucleation process also can be controlled, which results in some environmental and technological benefits, including the single-step production of crystalline materials, the energy-efficient process and the homogeneous products compared to the conventional processing of solid-state materials. In addition, this method is suitable for producing hybrid materials, oxides and non-oxides with various morphologies, which enables researchers to scale up the production level. During the hydrothermal process, mass transport occurs, which leads to the densification of the reaction materials, mostly by a dissolution precipitation mechanism, resulting in porous materials with good mechanical properties [58]. Firstly, the as-synthesized CDs were characterized to determine their particle size and elemental composition. As shown in Figure S1a, the average particle size (diameter) of the CDs was found to be 12.08 nm, measured using DLS spectroscopy. Furthermore, FT-IR spectra showed O-H stretching at 3320 cm^−1^, C-H stretching at 2920 cm^−1^, C=O stretching at 1750 cm^−1^ and C-O stretching at 1160 cm^−1^, which corresponds to the CDs (Figure S1b). In addition, the elemental composition of CDs was observed using EDS, which confirmed the presence of C, N and O in CDs (Figure S1c). The composition and purity of the obtained materials (FC and FCCD) were assessed using the XRD and EDS techniques. The XRD patterns for FC and FCCD25 are displayed in Figure 1b,c. For FC, the diffractions could be well indexed to spinel FeCo_2_O_4_ phase, corresponding to 2*θ* of 18.9° (111), 31.2° (220), 36.7° (311), 38.4° (222), 44.6° (400), 55.5° (422), 59.2° (511) and 65.1° (440), which are in good agreement with previous reports in the literature [59,60]. The absence of other diffraction peaks reveals the phase purity of the synthesized materials and also demonstrates that precursor is completely decomposed to spinel FeCo_2_O_4_ after calcination. In Figure 1c, the presence of characteristic peaks for FC and the appearance of amorphous CD peaks substantiates the successful growth of FC, even in the presence of CDs. The successful synthesis was also confirmed in other CD ratios (FCCD5 and FCCD40) in which the diffraction peaks of CD and FeCo_2_O_4_ can be found (Figure S2).

**Figure 2:**
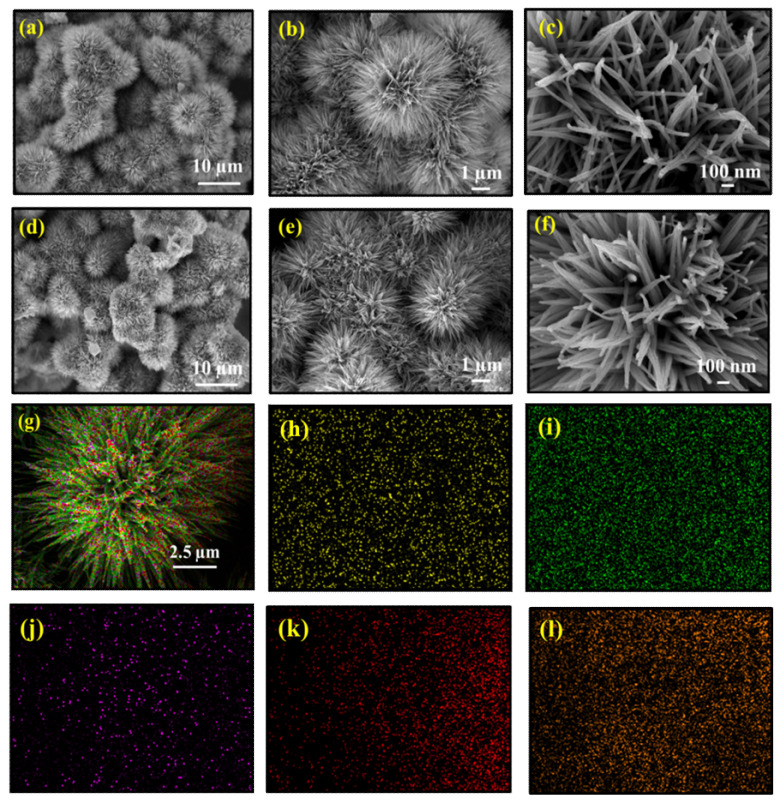
FESEM images of FC (**a**–**c**), FCCD25 (**d**–**f**) and nanoflower at different magnifications and (**g**) EDS mapping of FCCD25, showing the homogeneous distribution of Fe (**h**), Co (**i**), carbon (**j**), oxygen (**k**) and nitrogen (**l**).

The detailed morphologies and structural aspects of the as-synthesized materials were manifested by FESEM and TEM analysis. The FESEM images for FC and FCCD25 are represented in Figure 2, whereas the images for FCCD5 and FCCD40 are shown in Figure S3. From the images, FC and FCCD exhibit a nanoflower structure, which is mainly composed of interconnected nanowires. With the optimum loading of CDs (FCCD25), nanoflowers with bird’s nest-like cavities are formed (Figure 2d), but with a higher amount of CDs (FCCD40, Figure S3d–f), the structure becomes aggregated, which can hinder the electrochemical reactions. Thus, the judicious choice of CDs is of the utmost importance for controlling the morphology, which can regulate the electrochemical behaviors to a large extent. The elemental composition of FC and FCCD25 was studied using EDS (Figure S4 and Figure 2g–l). The corresponding mapping of FCCD25 demonstrates that it is chemically composed of Fe, Co, C, O and N, which are uniformly distributed across the Ni foam. In addition, Figure S5 also confirms the elemental composition of FCCD5, FCCD25 and FCCD40. This result clearly validates the successful incorporation of CDs in the nanohybrid.

**Figure 3:**
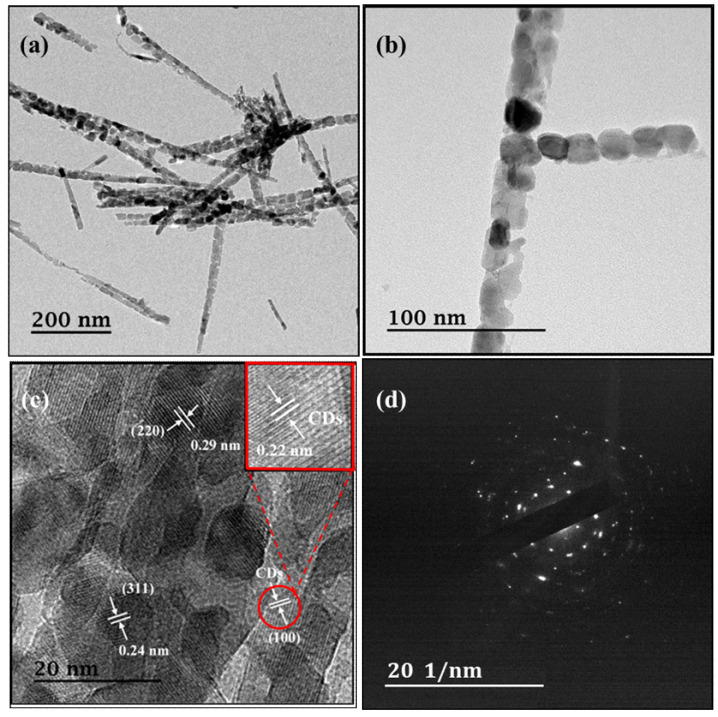
TEM (**a**,**b**), high-resolution transmission electron microscopy (HRTEM) (**c**) images and (**d**) selected area electron diffraction (SAED) patterns of FCCD25.

Further microstructure information is observed via TEM. TEM images for FC (Figure S6a,b) confirm the nanosheet-like structure. HRTEM analysis (Figure S6c) reveals lattice spacings of roughly 0.24 and 0.29 nm, indicating (311) and (220) lattices of FeCo_2_O_4_. Moreover, the selected area electron diffraction (SAED) (Figure S6d) indicates that the iron cobaltite formation corroborates the HRTEM and XRD data. The TEM images for FCCD25 are depicted in Figure 3a,b. It is obvious that FCCD25 exhibits a well-aligned structure, and the nanoparticles are interconnected, which can improve the Faradic reactions due to decreased ion transport path. From the TEM images, the length of the needle-like structure ranged from 0.8 to 1.0 μm, and the diameter of the needle was around 10–20 nm. The HRTEM image and SAED pattern (Figure 3c,d) clearly show lattice fringes of cubic FeCo_2_O_4_, indicating that the crystal growth is not disturbed in the presence of CDs. The lattice of 0.22 nm, which corresponds to (100) crystalline planes of CDs, is observed, confirming the successful doping of CDs into the FeCo_2_O_4_@CDs composite.

The surface area and the distribution of pore size in FCCD25 were characterized utilizing the BET method. The N_2_ adsorption and the pore volume distribution is presented in Figure S7 (with inset). The adsorption–desorption isotherm plot could be identified as a type-IV isotherm with a hysteresis loop ranging from 0.5 to 1.0, which reflects the formation of a mesoporous microstructure [33]. The total pore volume and the surface area was measured to be 0.179 cm^3^ g^−1^ and 52.20 m^2^ g^−1^, respectively. The distribution of pore size was calculated by the Barrett–Joyner–Halenda (BJH) method, which shows a wide range of pore size distribution between 3 and 30 nm, which arises from the spaces between the connected neighboring particles (FC and CDs) [40]. The mesoporous structure facilitates electrolyte diffusion, and the relatively large surface area increases the interfacial contact between electrode materials and electrolyte, which contributes to the significant enhancement of the electrochemical properties.

Further investigation of the elemental chemical state and compositions were conducted using high-resolution XPS (Figure S8). The survey scan revealed the presence of C, N and O from CD and Fe and Co from FeCo_2_O_4_ (Figure S8a). Prominent peaks at 711.6 eV and 724.5 eV (Figure S8b) are found, along with two weak satellite peaks at 717.5 and 733.4, which indicates the presence of Fe 2p_3/2_ and Fe 2p_1/2_, respectively. This result confirms the existence of Fe^3+^ in the spinel phase of FeCo_2_O_4_ [33,60,61]. The core level of Co 2p shows that the peaks centered at 780.8 eV and 795.1 eV arise due to 2p_3/2,_ and the Co 2p_1/2_ can be subdivided into four peaks after fitting, along with two satellite peaks (Figure S8c). The fitted peaks at 779.4 and 794.6 eV, along with the satellite peak at 787.7 eV, correspond to the Co^3+^, while the fitted peaks at 781.1 and 795.8 eV, along with the satellite peak at 804.3 eV, are indexed to Co^2+^. The spin-orbit splitting is calculated to be ~15 eV, signifying that these peaks are attributed to Co 2p_3/2_ and Co 2p_1/2_ and hence confirming the existence of Co^3+^ and Co^2+^ in the FeCo_2_O_4_@N-doped CDs [40,60]. Therefore, these XPS results reveal that FeCo_2_O_4_@N-doped CDs possess a composition consisting of Fe^3+^, Co^2+^ and Co^3+^. The deconvoluted C1s spectrum (Figure S8d) and the peaks at 284.4, 284.9, 285.5, 286.3 and 288.4 eV were associated with the C=C, C–OH, C–O/C=N, C–O–C and O–C=O bonds [62,63]. The O 1s spectrum consisted of a peak at 529.5 eV, which was ascribed to the metal–oxygen bond; the peaks at 530.8 and 532.1 eV reveal residual oxygen-containing surface functional groups of the CDs and physisorbed and chemisorbed water onto and within the surface (Figure S8e) [33,60]. The N1s spectra can be fitted into three peaks, which appear in the binding energy region at 398.9, 400.3 and 401.7 eV and can be attributed to the pyridinic N, pyrrolic N and graphitic N, respectively (Figure S8f) [64]. Thus, the presence of C1s, O1s and N1s peaks in the FeCo_2_O_4_@N-doped CDs (Figure S8d–f) signify the successful incorporation of CDs into the synthesized materials.

**Figure 4:**
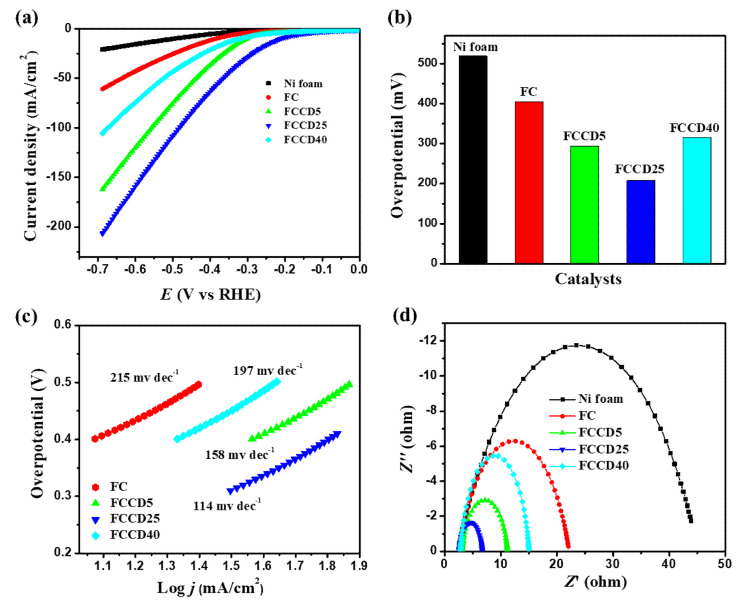
(**a**) Hydrogen evolution reaction (HER) polarization curves, (**b**) overpotential histogram, (**c**) Tafel plots and (**d**) Nyquist plots of different electrocatalysts.

After the successful characterization of the synthesized materials, we explored their electrocatalytic activity towards HER and OER. Nickel foam (NF) has been utilized as a template for synthesis due to its higher surface area, three-dimensional porous structure and electrical conductivity, and hence it can be utilized as an electrode substrate for probing the electrocatalytic activity of synthesized materials. A blank NF (in the absence of a catalyst) was used to probe the background electrochemical activities and demonstrated insignificant OER and HER catalytic activities. Generally, materials synthesized on NF showed improved performance towards HER and OER in the case of overpotentials and their current densities. We evaluated the HER performance of different electrocatalysts (FC and FCCD) by linear sweep voltammetry (LSV) measurements in order to examine the effect of CDs on the electrocatalytic activities. The HER polarization curves for the electrocatalysts are shown in Figure 4a and Figure S9a, and it is clear that FeCo_2_O_4_ with a CD loading of 25 mg (FCCD25) demonstrates the best HER activity compared to all the electrocatalysts. A current density of 10 mA cm^−2^ was reached, corresponding to HER at an overpotential of 205 mV for FCCD25, which is much lower than other electrocatalysts (Figure 4b), reflecting its superiority over all the electrocatalysts. A Tafel slope of 114 mV dec^−1^ was attained for FCCD25, which was eventually lower than those of other samples, rendering favorable kinetics for FCCD25 (Figure 4c and Figure S9b). In addition, commercially available Pt/C on the NF electrode was used to perform a control experiment within the experimental condition (Figure S9c,d). The Tafel slope of 38 mV dec^−1^ was obtained for the Pt/C in the HER experiment, which is consistent with the previously reported results [65]. The Tafel slope value indicates that HER proceeds via the Volmer–Heyrovsky mechanism [66]. The EIS measurements of different electrocatalysts was performed to assess the interface charge mobility of the electrode. From the Nyquist plots (Figure 4d), the lowest *R_ct_* was achieved for FCCD25, validated by the faster charge transfer kinetics, suggesting improved electrical conductivity. Additionally, *C_dl_* was determined to calculate ECSA (Figure S10). Based on these *C_dl_* values and the specific capacitance of material per unit area (*C_s_*, which is normally between 20 and 60 µF cm^−2^ for a flat surface), ECSA was calculated (Table S1) [67]. The *C_dl_* value for FCCD25 was 4.25 mF cm^−2^, corresponding to an ECSA of 106.25 cm^2^. The ECSA value of FCCD25 is 6.53, 2.36 and 2.93 times higher than those of FC, FCCD5 and FCCD40, illustrating efficient adsorption and reactant transfer to improve the electrochemical reaction in the case of FCCD25.

**Figure 5:**
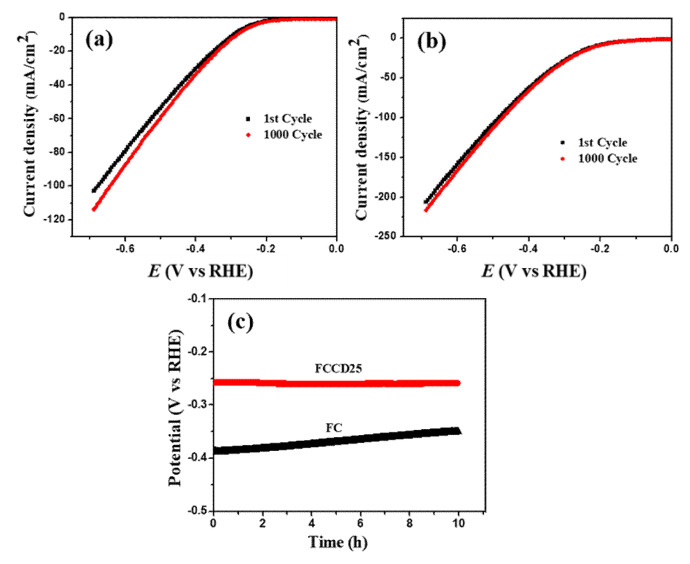
HER polarization curves of (**a**) FC, (**b**) FCCD25 before and after 1000 CV cycles, (**c**) chronopotentiometric study at a constant current density of 10 mA cm^−2^.

The long-term stability of catalysts is also important and becomes a key feature for water reduction and oxidation processes. To investigate this factor in terms of the HER process, 1000 CV cycles were measured on the FC and FCCD25 electrodes. Figure 5a shows that after 1000 CV cycles, the polarization curve remains the same at a low current density region for FC, but it deviates in a high current density region. However, for FCCD25 (Figure 5b), the polarization curve almost coincides with the initial one. Furthermore, FCCD25 exhibits excellent electrochemical durability over 10 h (Figure 5c) compared to FC. Figure S11 shows the XRD patterns and SEM images of FCCD25, which reveal no distinct change in the crystal structure of FCCD25 after 10 h chronopotentiometric study. The above results clearly demonstrate the pivotal role of CDs in providing the electrochemical stability to the electrocatalyst.

**Figure 6:**
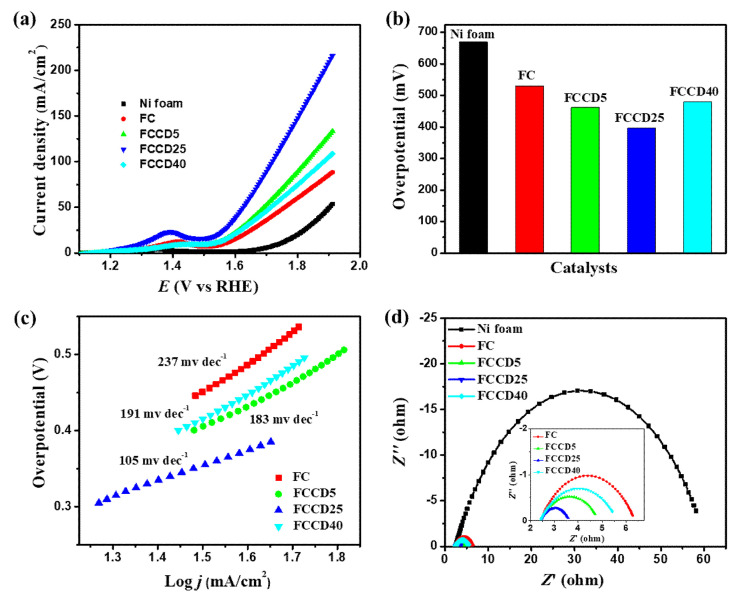
(**a**) OER polarization curves, (**b**) overpotential histogram, (**c**) Tafel plots and (**d**) Nyquist plots of different electrocatalysts.

All the electrocatalysts grown on Ni foam were checked for their OER activities (electrolyte: KOH 1 M). Figure 6a shows that FCCD25 possessed a much higher activity than other electrocatalysts. A strong peak of Co^3+/^Co^4+^ (1.25–1.5 V) can be found in Figure 6a, which is more prominent for FCCD25, implying that FCCD25 can facilitate the oxidation and promote the OER process [68]. To generate a current density of 50 mA cm^−2^, FCCD25 required an overpotential up to 393 mV, while other electrocatalysts needed much higher overpotentials (Figure 6b). Furthermore, the Tafel slope for FCCD25 was found to be 105 mV dec^−1^ (Figure 6c). In contrast, a high Tafel slope was obtained for FC (237 mV dec^−1^), FCCD5 (183 mV dec^−1^) and FCCD40 (191 mV dec^−1^). Therefore, it is obvious that FCCD25 demonstrates the best OER activity due to its smaller overpotential, with a higher rate of OER, which is reflected from its small Tafel slope. The higher ECSA value of FCCD25 (Table S1) compared to the others could facilitate the penetration of electrolytes through the working electrode, hence confirming the improved interfacial contact towards the electrolyte and enhancing the surface reactions [69]. As the OER process is also related to the *R_ct_* value, EIS spectra for different electrocatalysts were tested. From Figure 6d and Table S1, FCCD25 exhibits the lowest *R_ct_* value (1.15 Ω), whereas others show higher values, confirming the better OER activity of FCCD25.

**Figure 7:**
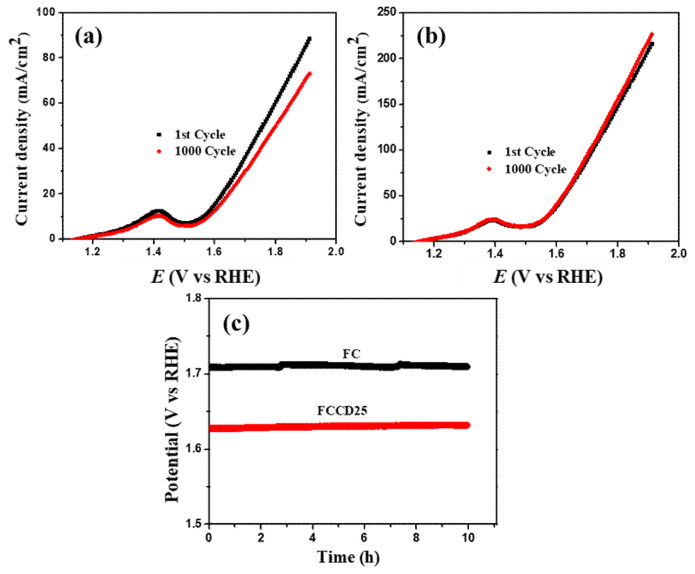
Oxygen evolution reaction (OER) polarization curves of (**a**) FC, (**b**) FCCD25 before and after 1000 CV cycles, (**c**) chronopotentiometric study at a constant current density of 50 mA cm^−2^.

FCCD25 electrocatalyst exhibits improved electrochemical stability compared to FC (Figure 7), which is manifested in the 1000 CV cycles test and the chronopotentiometric study. This superior activity and stability of FCCD25 towards OER is due to the synergistic effect between nanostructured FeCo_2_O_4_ and 0D CDs, which promotes the electrochemical reaction to a certain extent by rendering more exposed active sites. The overall water splitting performance of FC||FC and FCCD25||FCCD25 were evaluated and the results are presented in Figure S12 [70,71]. It is evident that FC||FC reaches 10 mA cm^−2^ at 1.64 V, whereas for the FCCD25||FCCD25, it takes 1.79 V, suggesting the better electrocatalytic performance of FCCD25. Moreover, the Mott–Schottky plot was recorded to investigate the reason behind the enhanced electrocatalytic activity of the FCCD25 sample by determining the flat-band potentials of the electrodes (Figure S13). The Mott–Schottky plots of FC and FCCD25 presented a negative slope in the linear region, which confirms that both FC and FCCD25 are p-type materials. The extrapolation of the linear part intersects with the voltage axis and provides the values of flat-band potential or the Schottky barrier, which was found to be 0.91 and 1.03 V for FC and FCCD25, respectively. The more positive flat-band potential of FCCD25 makes it a better catalyst by increasing the bandgap and conductivity [72]. Based on electrochemical measurements and the Mott–Schottky results, CD improves the electrochemical surface area and flat-band potential, which improves the electrocatalytic performance of the hybrid material. From the Tafel plot, FCCD25 has a smaller Tafel slope compared to FC due to the presence of CD, which indicates a faster charge transfer in the electrocatalytic process, as illustrated in Scheme S1. In addition, Table S2 shows the comparison of HER and OER performance between FCCD25 and previously reported materials [73,74,75,76,77,78,79,80,81,82]. Compared to HER active catalysts, FCCD25 showed a significantly lower overpotential. Additionally, FCCD25 showed better OER performance compared to other OER active catalysts, indicated by the lower overpotential. Thus, the present study demonstrates that the synthesized nanohybrid material can be useful for both hydrogen and oxygen evolution reactions.

## 4. Conclusions

FeCo_2_O_4_@N-doped CD samples were successfully synthesized through the hydrothermal method and utilized as a binder-free electrode for the electrocatalysis of water. The electrocatalyst with the optimum loading of CDs exhibits higher HER and OER activity compared to the other samples. The higher reactivity and electrochemical durability of FeCo_2_O_4_@N-doped CDs in comparison to the bare FeCo_2_O_4_ electrode result from the number of surface-active sites provided by the CDs. The active sites and the interaction of CDs with FeCo_2_O_4_ alleviate the charge transfer, which in turn improves the electrochemical reactions. We believe that this simple synthesis strategy will be useful for the fabrication of efficient electrocatalysts for suitable applications.

## Supplementary Materials

The following are available online at https://www.mdpi.com/1996-1944/13/14/3119/s1, Figure S1: DLS, FT-IR and EDS spectra of CDs, Figure S2: XRD pattern of FCCD5 and FCCD40, Figure S3: FESEM images of FCCD5 and FCCD40 nanoflowers at different magnifications, Figure S4: EDS mapping of FeCo_2_O_4_, Figure S5: EDS elemental percentage of FCCD5, FCCD25 and FCCD40, Figure S6: TEM, HRTEM images and SAED pattern of FeCo_2_O_4_, Figure S7: BET measurement of FCCD25, Figure S8: XPS spectra of FCCD25, Figure S9: HER polarization curve and Tafel plots for FCCD15, FCCD30 and Pt/C, Figure S10: the half differences in current density (*Ja-Jc*) plot against the scan rate, Figure S11: XRD and SEM images of FCCD25 after 10 h chronopotentiometric study, Figure S12: overall water splitting polarization curve of FC||FC, FCCD25||FCCD25 5 mV s^−1^ (without *iR* correction), Figure S13: Mott–Schottky plot of FC and FCCD25, Scheme S1: Illustration of charge transfer kinetics between FC and FCCD25, Table S1: Electrochemical parameters for different electrocatalysts, Table S2: Performance comparison for different electrocatalysts compared to FCCD25.
