# Supplementary material for: Construction of FeCo2O4@N-Doped Carbon Dots Nanoflowers as Binder Free Electrode for Reduction and Oxidation of Water
Source: Materials (Basel). 2020 Jul 13;13(14):3119. doi: 10.3390/ma13143119 (PMC7411927; doi:10.3390/ma13143119)
Supplement: Supplementary file 1 [file materials-13-03119-s001.pdf]

Supplementary Materials

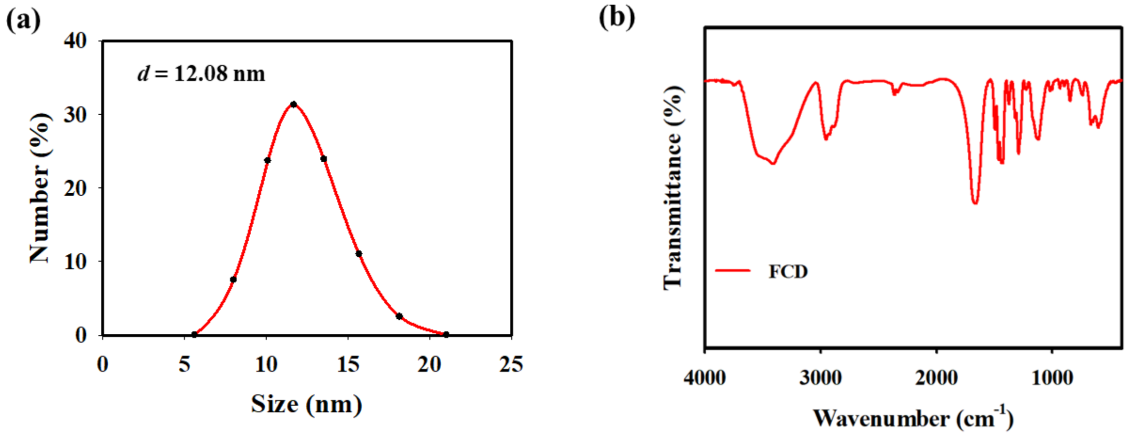

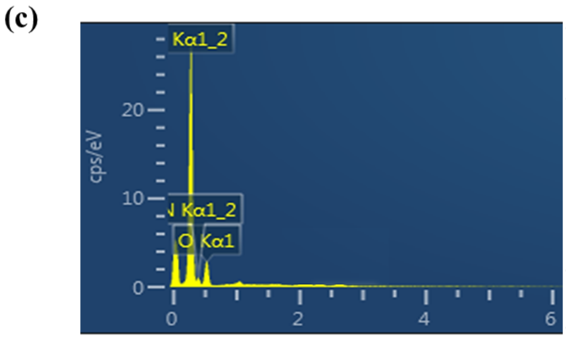

Element	*Wt*%	*Wt*% sigma
C	71.44	1.63
N	10.48	1.73
O	18.08	0.62
Total	100	

Figure S1. (**a**) DLS, (b) FT-IR, and (**c**) EDS spectra of CDs.

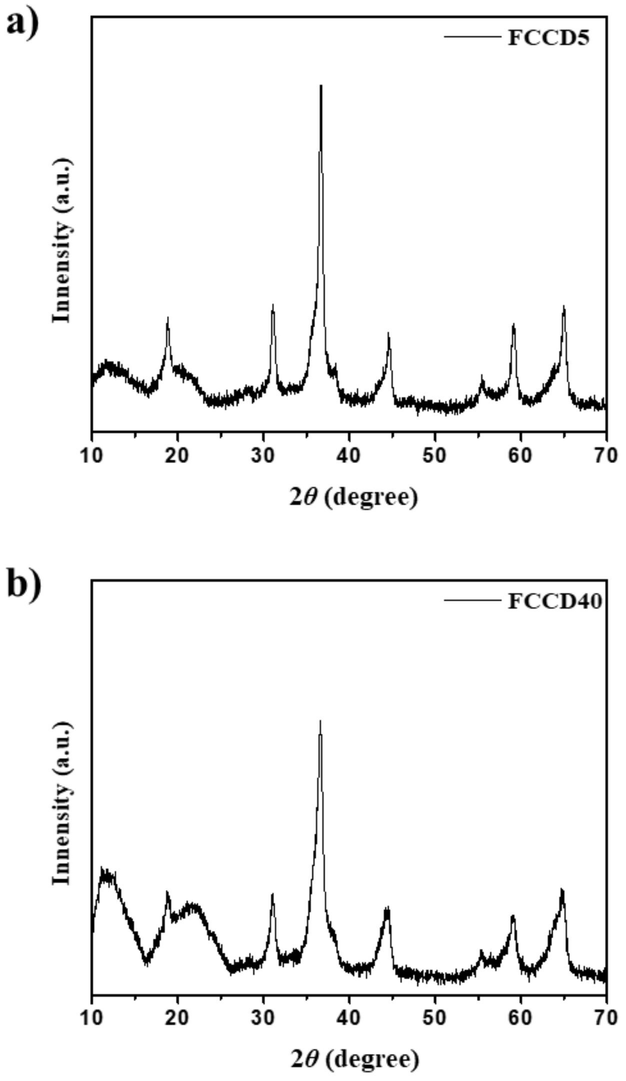

Figure S2. XRD pattern of FCCD5 (**a**) and FCCD40 (**b**).

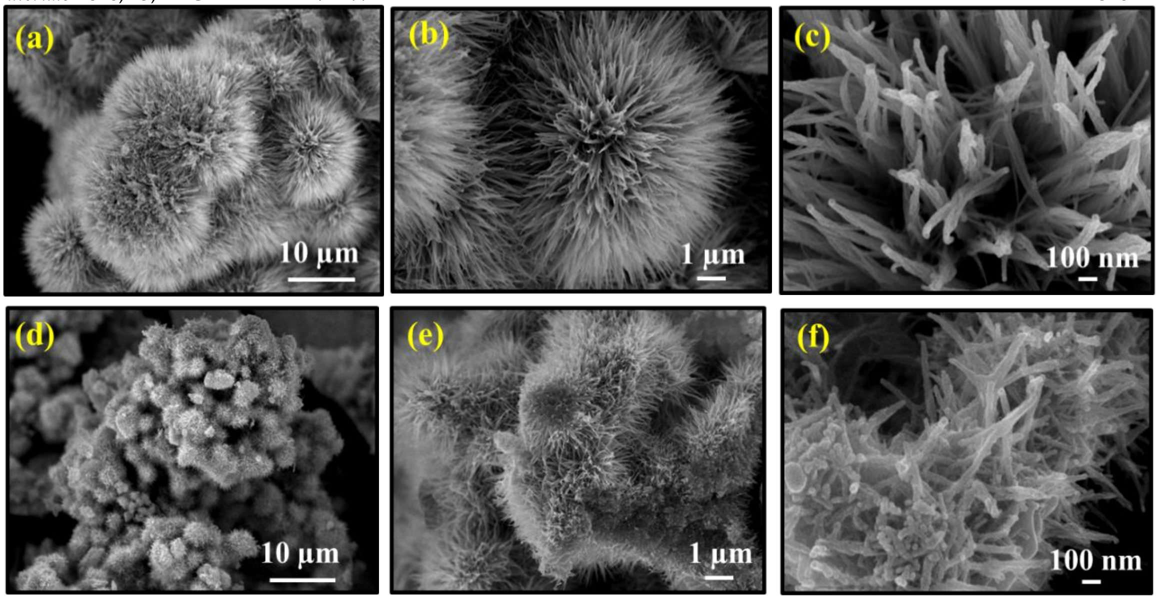

Figure S3. FESEM images of FCCD5 (**a**–**c**), FCCD40 (**d**–**f**) nanoflower at different magnifications.

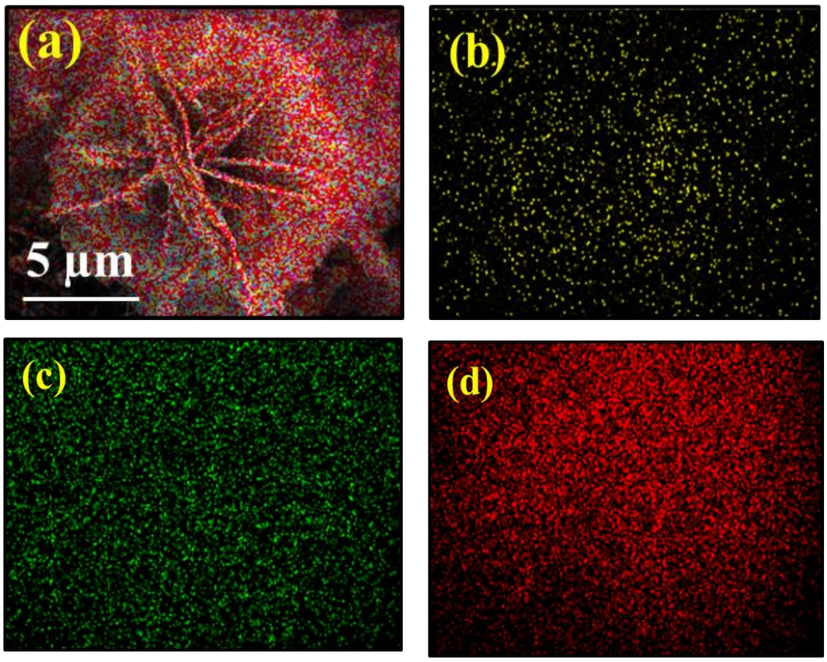

Figure S4. (**a**) EDS mapping of $FeCo_2O_4$ showing the distribution of Fe (**b**), Co (**c**), and oxygen (**d**).

a)

FCCD5

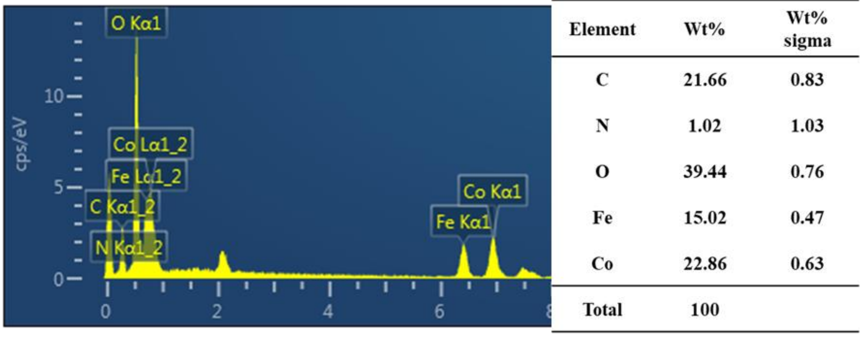

Element	Wt%	Wt% sigma
C	21.66	0.83
N	1.02	1.03
O	39.44	0.76
Fe	15.02	0.47
Co	22.86	0.63
Total	100	

b)

FCCD25

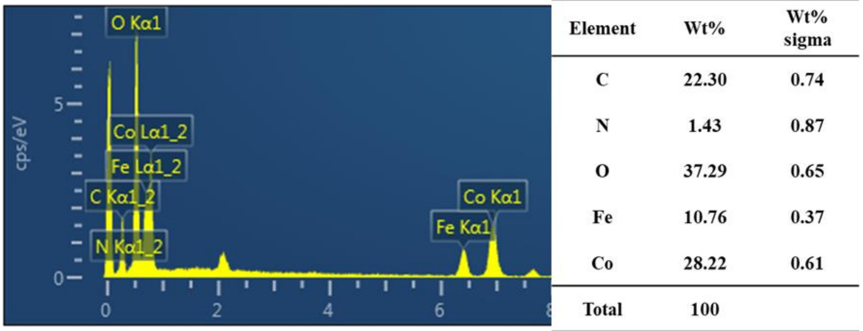

Element	Wt%	Wt% sigma
C	22.30	0.74
N	1.43	0.87
O	37.29	0.65
Fe	10.76	0.37
Co	28.22	0.61
Total	100	

c)

FCCD40

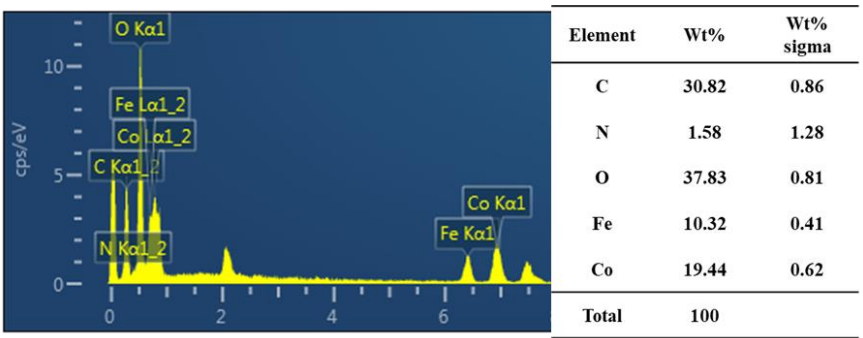

Element	Wt%	Wt% sigma
C	30.82	0.86
N	1.58	1.28
O	37.83	0.81
Fe	10.32	0.41
Co	19.44	0.62
Total	100	

Figure S5. EDS elemental percentage of (**a**) FCCD5, (**b**) FCCD25 and (**c**) FCCD40.

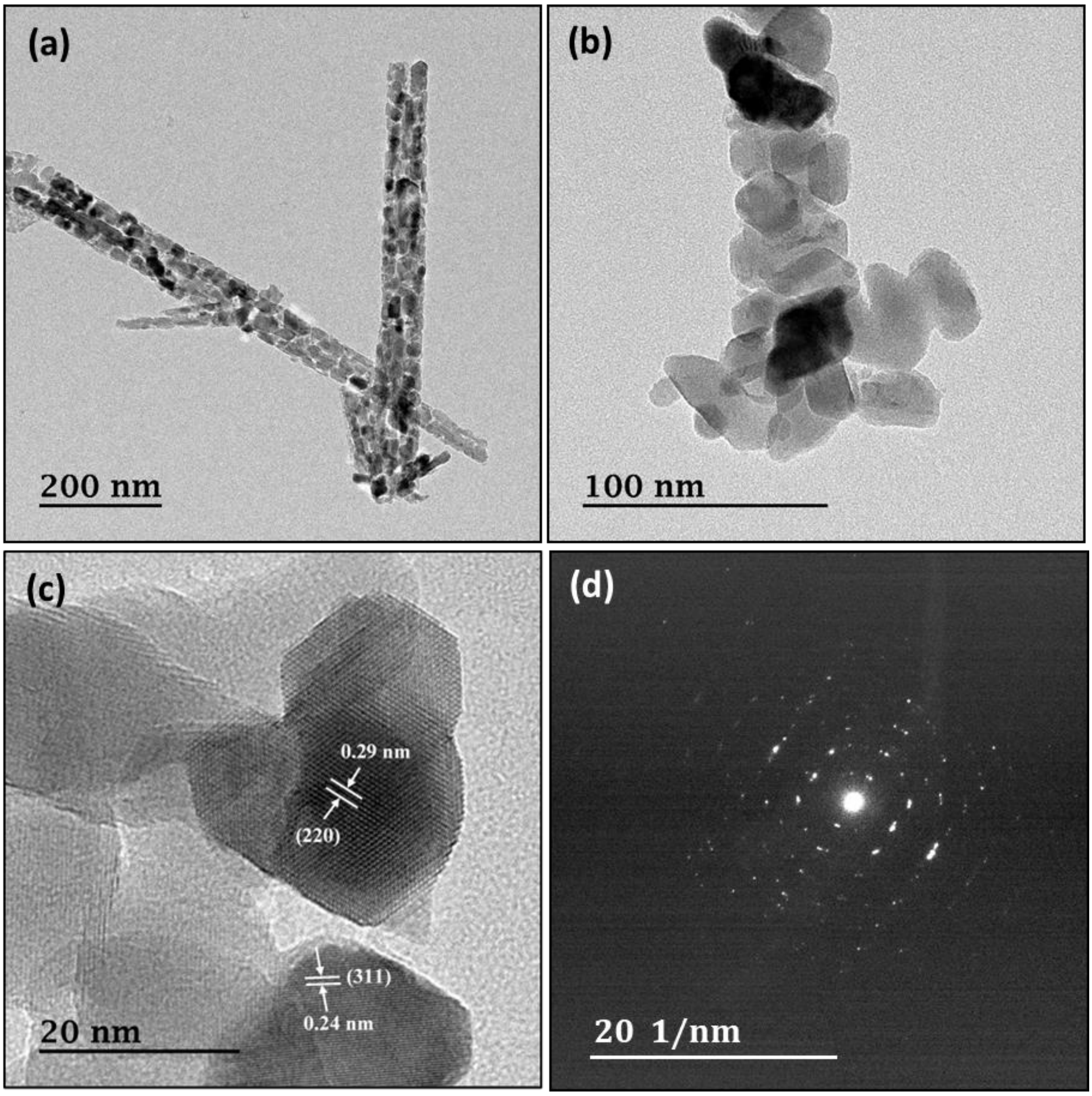

Figure S6. (**a**, **b**) TEM, (**c**) HRTEM images and (**d**) SAED pattern of $FeCo_2O_4$.

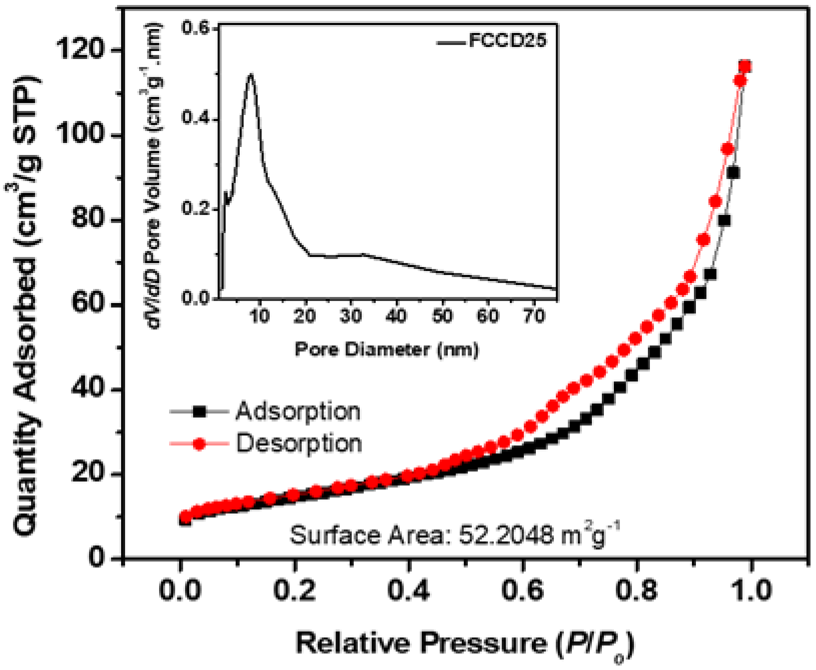

Figure S7. BET measurement of FCCD25. Inset: pore size distribution of FCCD25.

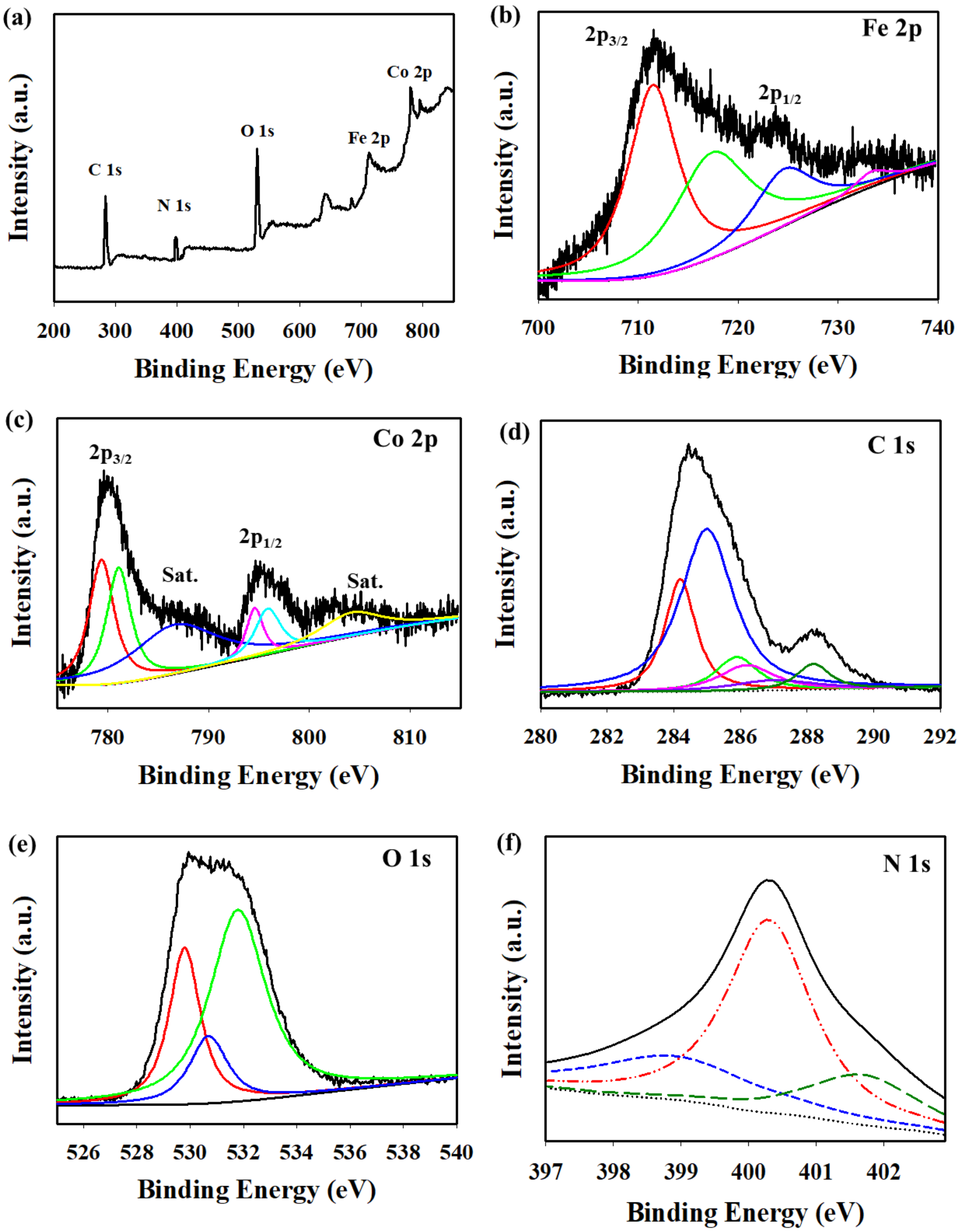

Figure S8. (**a**) survey scan spectra and (**b**–**f**) narrow scan spectra of Fe 2p, Co 2p, C1s, O1s and N1s for FCCD25.

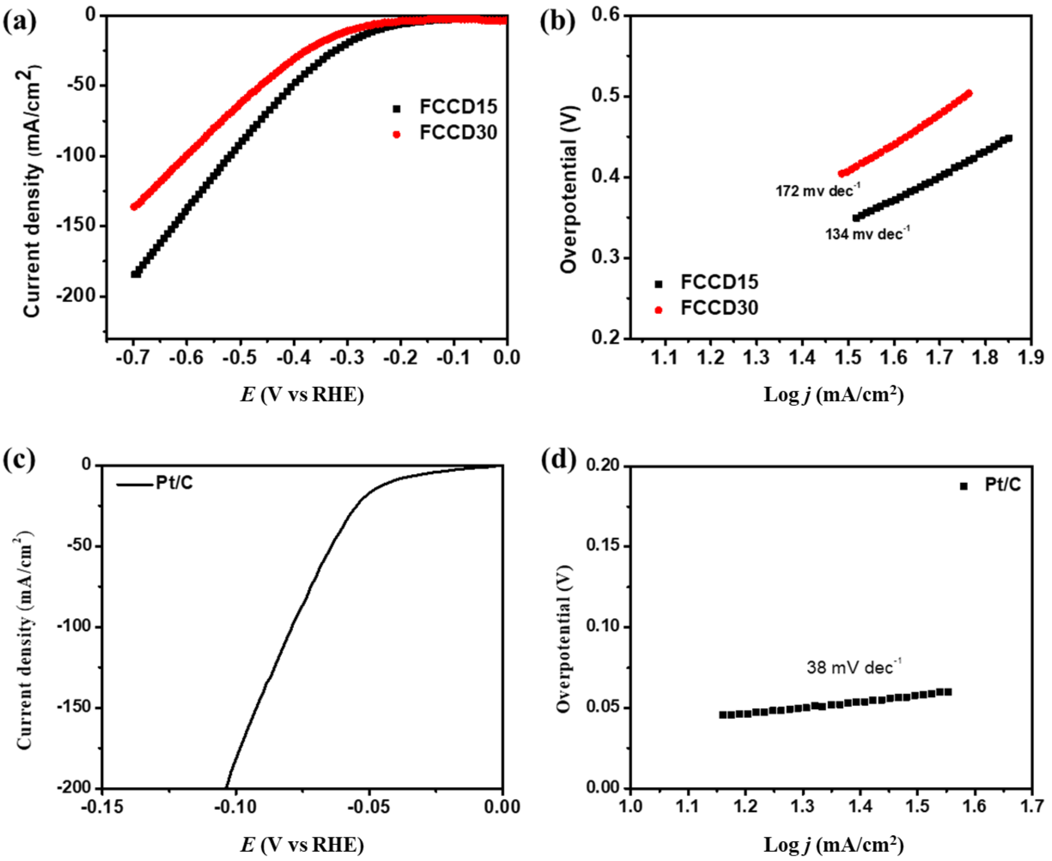

Figure S9. (**a**) HER polarization curve and (**b**) Tafel plots for FCCD15 and FCCD30. (**c**) HER polarization curve and (**d**) Tafel plots for Pt/C

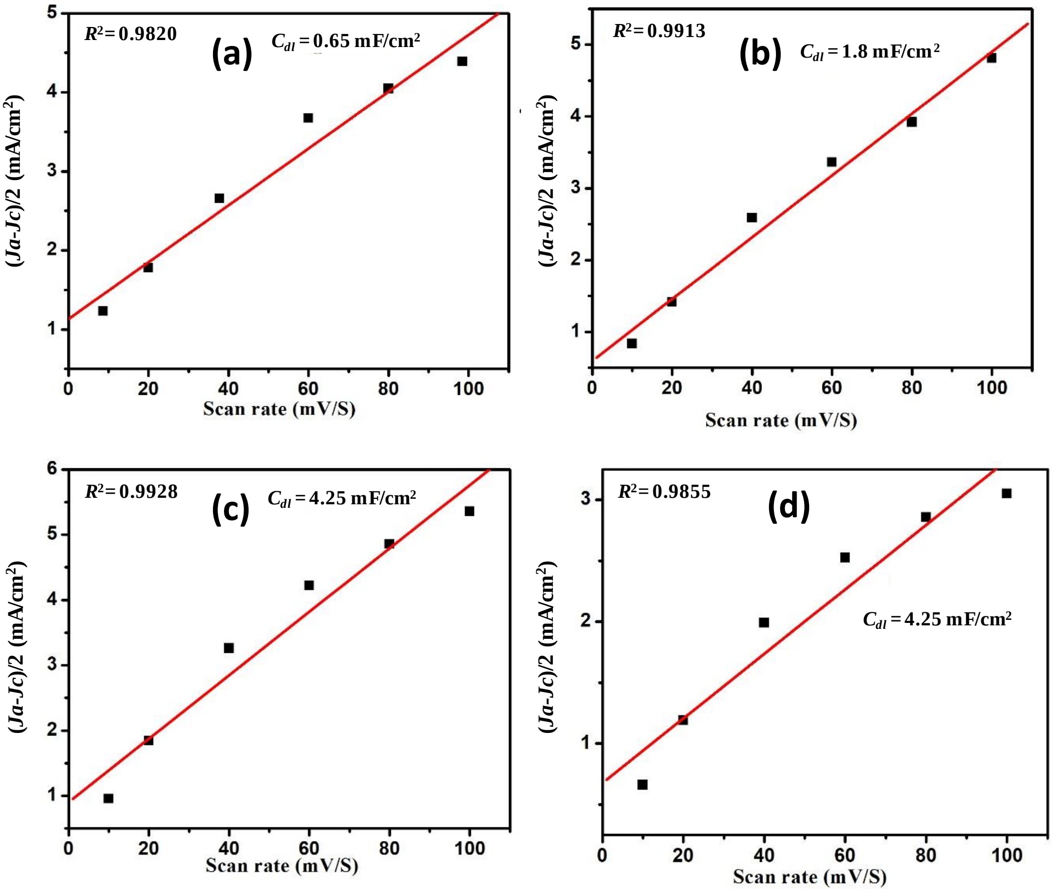

Figure S10. The half differences in current density (*Ja-Jc*) against scan rate for (**a**) FC, (**b**) FCCD5, (**c**) FCCD25 and (**d**) FCCD40.

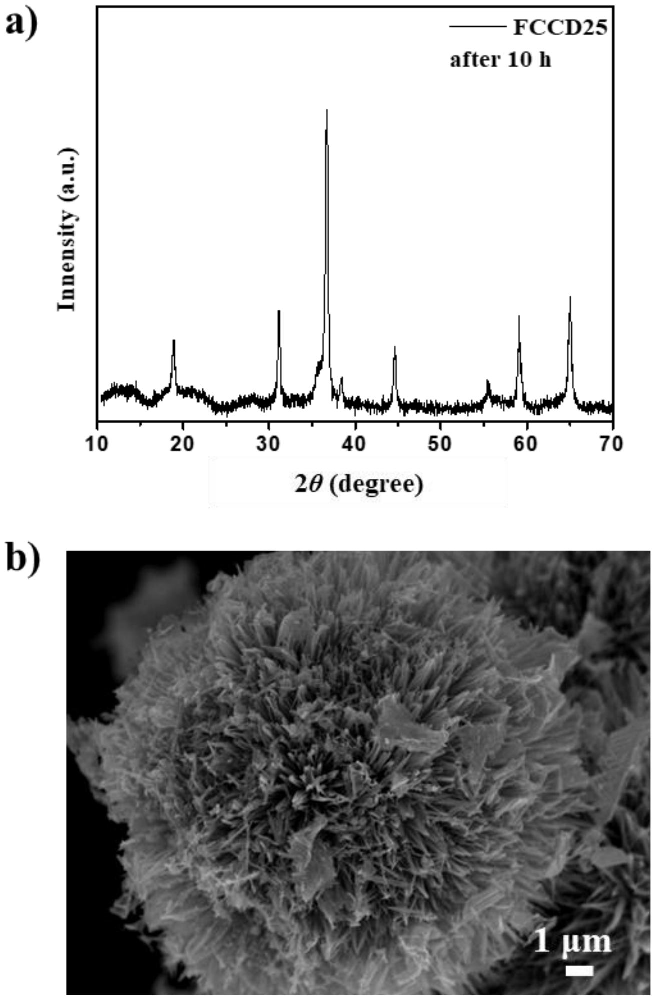

Figure S11. (**a**) XRD and (**b**) SEM image of FCCD25 after 10 h chronopotentiometric study.

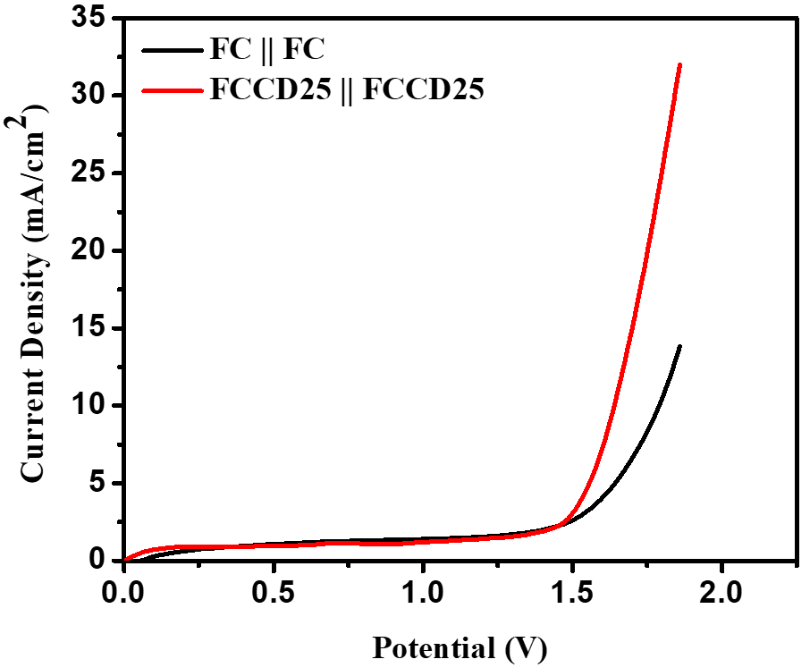

Figure S12. Overall water splitting polarization curve of FC||FC, FCCD25||FCCD25 5 mV s^{-1} (without *iR* correction).

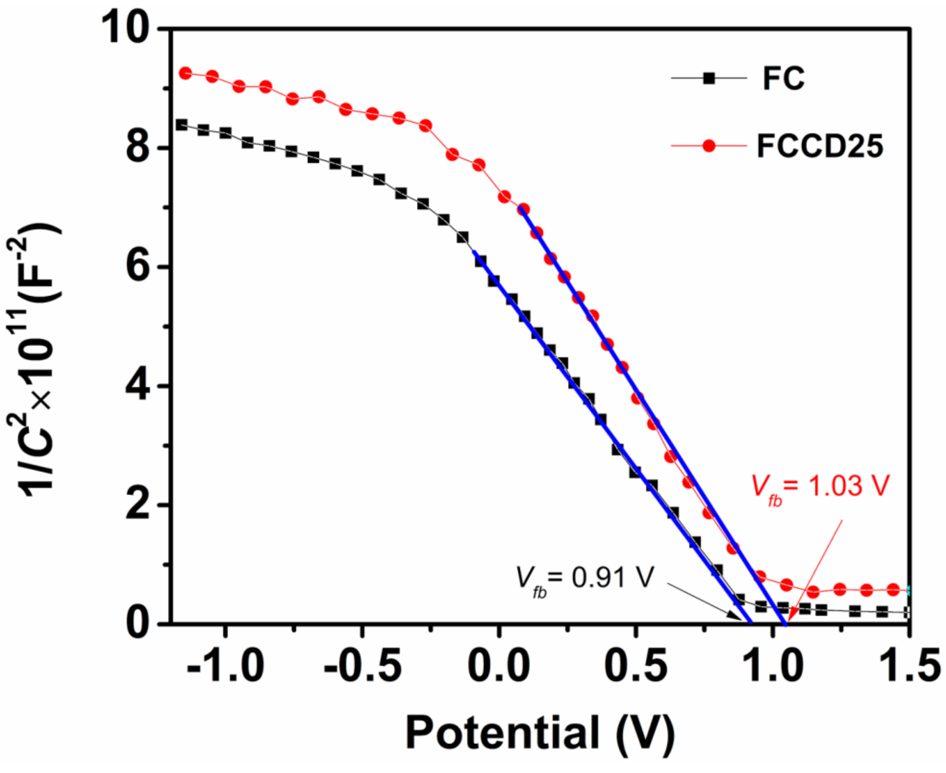

Figure S13. Mott-Schottky plot of FC and FCCD25.

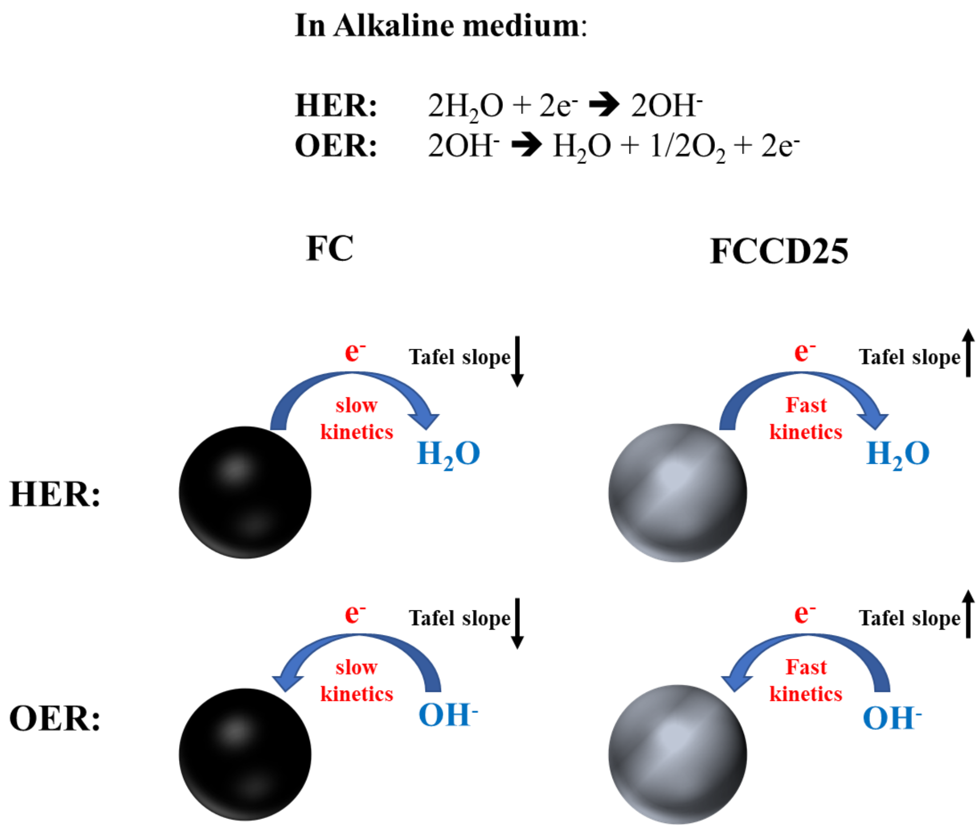

Scheme S1. Illustration of charge transfer kinetics between FC and FCCD25.

Table S1. Electrochemical parameters for different electrocatalysts.

Materials	η (mV)		Tafel slope (mVdec^{-1})		*Cdl* (mF/cm^2)	ECSA (cm^2)	R_{ct} (ohm)	
	HER η_{10}	OER η_{50}	HER	OER			HER	OER
FC	405	529	215	237	0.65	16.25	19.8	3.9
FCCD5	294	461	158	183	1.8	45	11.2	3.6
FCCD25	205	393	114	105	4.25	106.25	2.11	1.15
FCCD40	315	481	197	191	1.45	36.25	6.92	2.33

Table S2. Performance comparison for different electrocatalysts compared to FCCD25.

Electrocatalyst	Activity	HER overpotential (mV)	OER overpotential (mV)	Ref.
FCCD25	HER/OER	205	393	This Work
Ni_3N bulk	OER	-	490	[73]
$Ni_xCo_{3-x}O_4$	OER	-	530	[74]
$CuCo_2O_4$/NrGO	OER	-	410	[75]
Fe/N-CNTs	OER	-	520	[76]
$CoFe_2O_4$/rGO	OER	-	540	[77]
EG/$Co_{0.85}$ Se/NiFe LDH	HER	260	-	[78]
$CoSe_2$ nanocrystal	HER	450	-	[79].
Sm_2O_3-Ni-Co/NF	HER	276	-	[80]
Co_3O_4-NCTs	HER	370	-	[81]
CeO_2/Co(OH)$_2$	HER	317	-	[82]
